# Effect of the Olive Oil Extraction Process on the Formation of Complex Pectin–Polyphenols and Their Antioxidant and Antiproliferative Activities

**DOI:** 10.3390/antiox10121858

**Published:** 2021-11-23

**Authors:** Alejandra Bermúdez-Oria, Elisa Rodríguez-Juan, Guillermo Rodríguez-Gutiérrez, África Fernández-Prior, Juan Fernández-Bolaños

**Affiliations:** Department of Food Phytochemistry, Instituto de la Grasa (Spanish National Research Council, CSIC), Pablo Olavide University, Building 46, Ctra de Utrera km 1, 41013 Seville, Spain; aleberori@ig.csic.es (A.B.-O.); elisarodriguez@ig.csic.es (E.R.-J.); guirogu@ig.csic.es (G.R.-G.); mafprior@ig.csic.es (Á.F.-P.)

**Keywords:** phenols–pectic–polysaccharides complex, antioxidant activity, olive oil, alperujo, cell wall material, Caco-2 cell line

## Abstract

The aim of this research was to investigate the interaction of phenols and pectic polysaccharides during the olive oil extraction process. For this, pectin was extracted from fresh olive fruits and compared to the pectin isolated from the paste resulting from the extraction of the olive oil after milling with malaxation at 30 °C/30 min and subsequent centrifugation of the olive paste from the same lot of olive fruits in a system called ABENCOR (AB). The results indicate that these interactions were enhanced during the olive oil extraction process. In addition, the resulting AB extracts exhibited high antioxidant activity (ORAC) and strong antiproliferative activity in vitro against colon carcinoma Caco-2 cell lines compared to olive fruit extracts. The polyphenols associated mainly with the acidic pectin substance, with a higher content in AB extracts, seem to be responsible for these activities, and appear to maintain their activities in part after complexation. However, even in olive fruit extracts with smaller amounts of phenols in their compositions, pectic polysaccharides may also be involved in antioxidant and antiproliferative activities.

## 1. Introduction

Olive fruit is widely consumed in the world. The production of olives is around 19 million tons, with 90% being consumed as olive oil and 10% as table olives [1]. Nowadays, olive industries produce a large amount of waste and present an environmental problem. Alperujo is a by-product generated from the two-phase extraction process of olive oil. In fact, the annual production of alperujo in Spain alone reaches 4–6 million tons, and this poses a challenge for its disposal because of its high amounts of organic matter and phytotoxic compounds. However, alperujo is a promising source of substances with high added value, including phenolic compounds, which represent 98% of the total phenols in the olive fruit, while the remaining 2% (these include hydroxytyrosol, tyrosol, verbascoside, oleuropein secoiridoid structures, hydroxytyrosol and tyrosol bound to the dialdehydic form of elenolic acid and flavonoids) confer the bioactive properties of olive oil [2]. Another important component of alperujo is the cell wall material that is mainly composed of cellulose, xylan, glucurunoxylan, xyloglucan and pectic material, which is rich in arabinose [3,4]. In fact, pectic polysaccharides compose one-third of the olive cell walls [4].

Polyphenols that have the capacity to interact covalently (non-extractable polyphenols) or non-covalently (extractable polyphenols) with plant cell walls have been described previously [5,6]. Unlike extractable polyphenols, non-extractable polyphenols are not extractable by organic solvents. They are generally higher molecular weight polyphenols, with a complex structure that can form polymeric compounds or bind to carbohydrates or proteins [7,8]. Such interactions can modify the bioaccessibility, bioavailability and bioefficacy of polyphenols. In fact, the amount of phenolic and procyanidin compounds found in wine or apple and pear juices is lower than those found in whole fruits [9,10,11], mainly due to the formation of phenol-carbohydrates during processing. These bound polyphenols reach the colon where they are released and fermented by bacteria into absorbable metabolites that may be essential for maintaining good gut health [12,13].

In the case of olive fruit, during the rupture of fruit tissues by olive crushing and further malaxation at moderate temperature (milling and malaxation) of the olive paste before oil extraction, cells are ruptured and interactions take place between cell wall polysaccharides and hydrophilic compounds (phenols, proteins, etc.). Moreover, oxidation, condensation and/or polymerization reactions by enzymatic or nonenzymatic action provide an important non-carbohydrate polymeric material associated with cell-wall polysaccharides [14]. Capasso, de Martino, and Arienzo [15] found a polymeric structure called polymerin. It was recovered from olive oil mill wastewater that was made up of carbohydrates, melanin and proteins, as well as some cations, which were bound and chelated to said structure. Our previous studies have shown the presence of significant amounts of phenolic compounds bound to the pectic material isolated from steam-treated alperujo, with the phenolic compounds being mainly responsible for the important antioxidant and antiproliferative properties of these extracts [16].

Due to the great importance of the formation of non-extractable polyphenols by interaction between the polysaccharides in the cell wall and phenolic compounds, which only a minor part of the existing literature on polyphenols addresses, and based on the findings of our research group, the aim of the present study was to obtain better understanding of the mechanisms involved in the formation of such non-extractable polyphenol/carbohydrate bonds during the olive oil extraction process. Therefore, in this work, the changes in the composition and structure of these fiber-antioxidant extracts were studied in order to evaluate how the milling, malaxation and centrifugation of the olive affect its formation, as well as its relationship with antioxidant and antiproliferative activities. Firstly, extraction of the pectin from the fresh olive fruits was carried out. Secondly, it was compared to the pectin isolated from the paste from the extraction of the olive oil after milling, malaxation at 30 °C/30 min and subsequent centrifugation of the olive paste in a system called ABENCOR, which, in the laboratory, simulates the process of extraction of olive oil in the mill. In both cases, and prior to the extraction of pectins, the free phenols and all the material that is soluble in alcohol were eliminated by means of an exhaustive 80% ethanol wash.

## 2. Materials and Methods

### 2.1. Olive Fruits

The study was carried out with olives fruits (*Olea europaea* L.) of the Hojiblanca and Picual varieties, from trees growing in Pablo Olavide University (Seville, Spain). These samples were collected in January 2020.

### 2.2. Laboratory Scale Oil Extraction by ABENCOR System

Alperujo was obtained using the ABENCOR system (MC2 Ingenierías y Sistemas, Sevilla, Spain). The ABENCOR is a three-phase extraction system that serves to obtain olive oil samples on a laboratory scale in sufficient quantity to carry out different assays. In our research work, the ABENCOR system was used to obtain samples of the by-product of olive oil extraction.

The ABENCOR system involves a process of milling the olives until a paste is formed, which is then thermally beaten at 30 °C for 30 min. The by-products generated from the ABENCOR system are wastewater and pomace and, therefore, it is a three-phase system. The combination of wastewater and pomace phases produces a by-product similar to the one obtained at the industrial level in the pomace mills called alperujo (pomace).

### 2.3. Preparation of Cell Wall Material from Olive Fruit and ABENCOR System

Alcohol-insoluble solids (AIS) from olive fruits and ABENCOR system were prepared according to the method of Renard [17]. Briefly, the olives were pitted, 96% ethanol was added, and the mixture was held for 30 min. Subsequently, olive fruits and ABENCOR were directly ground in a domestic blender in 96% ethanol and subsequently filtered through a nylon cloth. The resulting solid was ground and washed repeatedly with 70% ethanol until the filtrate had no color. Drying was performed by solvent exchange (96% ethanol and acetone), then overnight in an oven at 40 °C. For AIS from ABENCOR, a sieve between 400 and 100 µm was used to separate the stones.

### 2.4. Isolation and Purification of Pectin from AIS

The AIS from olive fruits and ABENCOR were treated with citric acid (30 g AIS/300 mL 3% citric acid) for 60 min at 80 °C in a bath and 120 °C in autoclave in order to extract the pectins from the AIS.

All extracts were neutralized with NaOH (1M) to initial pH and centrifuged for 10 min at 16,000× *g* (Sorvall RC5C, DuPont, Nemours, Mechelen, Belgium). Subsequently, the extracts were dialyzed in 12,000 Da bags for 48 h and then freeze-dried. Finally, two extracts called Olive fruit and AB (obtained from ABENCOR system) were obtained.

### 2.5. Characterization of Pectin Extracts

#### 2.5.1. Uronic Acid Analysis

Galacturonan (anhydrogalacturonic acid) was determined according to the m-hydroxydiphenyl method described by Blumenkrantz and Asboe-Hansen [18] for uronic acids.

#### 2.5.2. Glycosyl Analysis

Glycosyl compositions were determined by gas chromatography (GC) after conversion to alditol acetates. Individual neutral sugars were analyzed from duplicate samples with initial TFA hydrolysis (2 N TFA at 121 °C for 1 h) prior to reduction, acetylation and analysis by GC [19] using inositol as internal standard. Calibration was performed using a series of standard solutions of L-rhamnose (Rha), L-arabinose (Ara), D-galactose (Gal), D-glucose (Glc), D-mannose (Man) and D-xylose (Xyl). The chromatographic conditions were described by Lama-Muñoz, Rodríguez-Gutiérrez, Rubio-Senent and Fernández-Bolaños [20].

#### 2.5.3. Total Phenolic Analysis

Total phenolic content was determined using the Folin–Ciocalteu spectrophotometric method and expressed as grams of gallic acid equivalents [21].

#### 2.5.4. Protein Analysis

The protein composition was determined with an elementary analyzer (LECO, St. Joseph, MI, USA) by the Dumas method, which consists of an internal combustion and thermoconductivity at 1000 °C. The protein content (g/100 g^−1^) was calculated using the conversion factor 6.25 (ISO, 2016) [22].

#### 2.5.5. Ash Analysis

The analysis of ash was performed according to the AOAC procedure [23] (AOAC, 1990, pp. 915–919).

### 2.6. Antioxidant Activity: Oxygen Radical Absorbance Capacity (ORAC) Assay

The ORAC assay is based on the inhibition of peroxyl radical-induced oxidation initiated by the thermal decomposition of 2,2′-azobis (2-amidino-propane) dihydrochloride (AAPH). The reactive oxygen species (ROS) produced diminishes the fluorescence signal generated by the fluorescein. The antioxidant capacity of samples was assayed according to Ou, Hampsch-Woodill, and Prior [24] with minor modifications. The samples were conveniently diluted with sodium phosphate buffer (10 Mm, pH 7.4), and 25 µL of sample were transferred to a microplate. A blank with 25 µL of phosphate buffer was used, while standards received 25 µL Trolox solutions (10–140 µM). Then, 150 µL of 1 µM fluorescein were added to all wells. After incubation (37 °C, 15 min), 25 µL AAPH (250 mM) were added to each well to initiate the reaction and a reading was taken every 5 min for 90 min (Ex. 485 nm, Em. 538 nm). Absorbance was measured in a microplate reader (Fluoroskan AscentTM, Thermo ScientificTM, Waltham, MA, USA). Results were calculated using the difference of areas under the fluorescein decay curve between the blank and the sample and were expressed as µmol Trolox equivalents/g of sample.

### 2.7. Relative Molecular Weight Determination

The homogeneity and molecular weight distribution of the purified extracts were estimated by high performance size-exclusion chromatography (HPSEC) using two different columns (300 × 7.8 mm i.d., Tosoh Bioscience LLC, King of Prussia, PA, USA) in sequence TSK gel GMPWXL (dextran Mw < 50,000 kDa) and TSKgel G3000PWXL (dextran Mw < 60 kDa), as described previously [25]. The system was calibrated with standard dextrans of 500, 110, 70, 40, and 6 kDa.

### 2.8. Cell Culture and Treatment

The colon cancer cell line (Caco-2) was provided by the European Collection of Authenticated Cell Cultures, Public Health England. Cell culture media and serum were from Gibco, Thermo Fisher Scientific (Waltham, MA, USA). Caco-2 cells were kept at 5% CO_2_ in Dulbecco’s Modified Eagle Medium (1000 mg/mL glucose, 110 mg/mL pyruvate, and 580 mg/mL glutamine) supplemented with 10% fetal bovine serum, 1% non-essential amino acids, 100 u/mL penicillin, and 100 μg/mL streptomycin. Fetal bovine serum was heat-inactivated at 56 °C for 30 min. Caco-2 cells were subcultured once a week using trypsin-ethylenediaminetetraacetic acid, and the medium was renewed once in between passages.

The freeze-dried extracts (Olive fruits and AB) were dissolved in Hank’s Balanced Salt Solution (HBSS) at 100 mg/mL, heated in a thermostatized bath at 90 °C for 20 min, and diluted with culture medium as required. Cells were seeded in 96-well microplates (4 × 10^3^ or 14 × 10^3^ cells/well, 50 µL/well). Extracts were added in the same volume (50 µL/well) to achieve a final concentration of 1.1 to 10 mg extract/mL DMEN, 10%HBSS. Cells without extracts were performed as control (DMEN, 10% HBSS). In addition to the cell viability assay as shown below, cells were inspected under the phase contrast microscope.

### 2.9. Cell Proliferation Assays

The proliferation of Caco-2 cells was determined by measuring viability at different times (1, 2, 4 and 7 days) using the neutral red assays. Cells in 96-well plates were incubated in a fresh culture medium containing the vital stain neutral red (50 μg/mL) for 30 min. Then, cells were washed with HBSS, and the neutral red was extracted using acetic acid (75 µL, 1% (*v*/*v*) in ethanol 50% (*v*/*v*)). Absorbance was measured in a Thermo Scientific Multiskan Spectrum spectrophotometer with a microplate reader by Thermo Fisher Scientific (Waltham, MA, USA) at 550 nm. [26,27].

### 2.10. Statistical Analysis

Results were expressed as mean values ± standard deviation. STATGRAPHICS^®^ plus software was used for statistical analysis. Comparisons among samples were made using one-way analysis of variance (ANOVA) and the LSD (Least Significant Difference) method. A *p*-value < 0.05 was considered significant

## 3. Results

### 3.1. Chemical Composition and Molecular Weight of Pectin-Polyphenol Extracts

Table 1 shows the extraction yield and chemical composition of the extracts obtained from the cell wall material (AIS) of fresh olive fruits and the alperujo generated from the same lot of fresh olive fruits by the ABENCOR system. Both AIS samples were subjected to conventional pectin extraction using 3% citric acid as the extractant agent at two temperatures, 80 and 120 °C, for 1 h, followed by purification with dialysis and freeze-drying. The compositional analysis of the olive fruit extracts and AB extracts revealed the presence of carbohydrates including uronic acid, and they also contained polyphenols, ash and a small amount of protein, mostly <5%. A significant difference was recorded in the case of phenolic compounds and protein contents. These differences increased with the effect of olive processing, doubling the amount of both proteins and phenols. Although the fresh olives were crushed with ethanol during the preparation of the AIS to remove free phenols (most localized inside the vacuole in intact olive cells), and to avoid their oxidation or condensation with the cell wall, the extracts obtained presented phenolic contents of 5.3 and 7.0 g/100 g extract for extraction at 120 and 80 °C, respectively. These contents would be partly explained by the presence of insoluble-bound phenolics, which are located in the cell wall matrix, through a vesicle transfer system, and are bound to macromolecules such as structural protein, cellulose and pectin through covalent bonds via ether, ester and carbon-carbon bonds [28,29]. The phenolic compounds content of the AB samples was double the amount present in the extracts of the fruit during the processing, with 12.0 and 15.5 g/100 g of extract for extraction of pectins at 120 and 80 °C, respectively. The contents in protein in the extracts varied in a similar way, doubling their percentages with processing. These increases are due to the crushing in the process of disruption of olive cell material, including the breaking of cell walls, and exposure of the matrix to enzymes, oxygen, and mild heat malaxation, which could be attributed mainly to condensation reactions and other intermolecular reactions between free phenols, protein and polysaccharides. These interactions, which are fast and spontaneous, occur systematically in fruit and vegetable processing [30]. During olive fruit processing and olive oil production, these conditions are ideal for these interactions to take place. In addition, the high affinity observed of the pectin with hydroxytyrosol (HT) and 3,4-dihydroxyphenylglycol (DHPG), the major antioxidant phenols found in olive fruit [31], may help explain the results found. Likewise, the addition of pectin-degrading enzymes during malaxation, which improves the phenolic content in virgin olive oils [32,33], may explain the degradation of pectin in the olive paste and the release of phenols in the olive oil.

Polysaccharides were also evaluated, and the compositional analysis of their saccharide part is shown in Table 2. In AB samples, the uronic content decreased 1.8-fold in the case of extraction at 80 °C and 1.3-fold at 120 °C compared to olive fruit samples. This fact was correlated with a higher content in phenolic compounds; when this occurred, the uronic acid content decreased. These polyphenols seem to be retained with the pectin in the form of conjugation and make extraction of this pectin difficult. This was demonstrated by analysis of the neutral sugars in the extracts. The analysis of monosaccharides showed that the polysaccharides were composed mainly of galactose, arabinose and rhamnose, together with uronic acids, suggesting the predominant presence of arabinogalactan associated with rhamnogalacturonan, the most common pectic sugar, whose composition was similar to the pectic polysaccharide previously reported by Cardoso et al. [34] for olive pomace. The amount of neutral sugar in the AB sample extract decreased by 1.7-fold in the samples at 80 °C, compared to olive fruit samples, in a similar proportion to uronic acid. These results showed that the extraction of the olive oil processing caused the phenolic compounds to be linked to the pectin, thus making them less extractable. In the case of the treatment of extraction at 120 °C, in both extracts, an important loss of arabinose occurred due to degradation at high temperatures. This behavior coincided with that of the extract at 80 °C, with a 1.3-fold decrease in uronic acid, which closely coincided with the 1.56-fold decrease in the sum of the amounts of rhamnose, arabinose and galactose, for the AB sample compared to the olive fruit sample. Furthermore, this finding suggested that the polyphenols can be associated with the pectin during the processing, and have a remarkable impact on their extraction. However, in this case, other sugars, such as xylose, mannose and glucose, showed an increase in their amounts, suggesting the presence of other types of polysaccharides (xylans, glucomannans, xyloglucans), which have been previously described as constituents of the olive pulp [3] and as being more easily extractable at higher temperatures.

Therefore, the phenolic compounds together with the proteins found in the extracts obtained from AIS might be linked to polysaccharide material through weak interactions, mainly hydrophobic, through the phenolic aromatic ring, reinforced by hydrogen bonding and/or even by stronger covalent interactions with the quinone formed from the oxidation of polyphenols (chemically or enzymatically mediated, e.g., polyphenol oxidase) [30], forming polyphenolic–protein–polysaccharide complexes, which are favored by olive processing.

**Figure 1:**
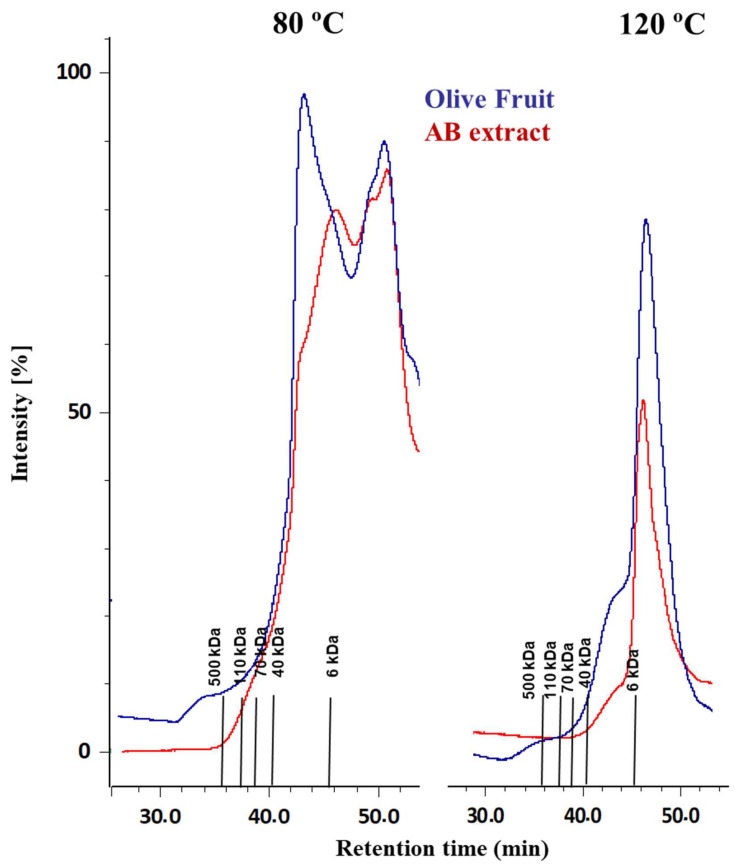
Refractive index elution profiles from Olive Fruit and AB extracts at 80 and 120 °C obtained by high-performance size exclusion chromatography with two TSK gel columns (GMPWXL and G3000PWXL) placed in a series. Lines represent molar masses of dextran.

Finally, the extracts were separated by size-exclusion chromatography and the molecular size of pectin–polyphenol complexes was estimated. The elution profiles of the extract of the fresh olive fruit at 80 °C shows a minor peak at about 500 kDa, which was not detected in the AB extract (Figure 1). Furthermore, fragments between 40 and 6 kDa from the olive fruit extract decreased slightly toward lower molecular weight ranges during processing (AB extract). A major peak with 6 kDa was observed for the AB extract elution profile, which coincided with the molecular weight (6 kDa) of the fragments of pectic polysaccharide solubilized with phosphate buffer from olive fruit [35]. Endogenous enzymes such as glycosidases and polygalacturonase [36,37] are expected to be activated in the ABENCOR process itself (milling, malaxation). Therefore, the slight decrease in molecular weight in processing samples took place.

Additionally, it was observed how the heat treatment at 120 °C for the extraction of the pectin–polyphenols complex affected their molecular weight, moving towards lower molecular weights in all the samples. For the olive fruit extract, the elution profile partly maintained its high molecular weights, although some decrease was observed with the appearance of a shoulder between 40 and 6 kDa and a major peak with a molecular weight lower than 6 kDa, which coincided with that of the AB extract. These results indicated that the molecular weight distribution of the pectin–polyphenols complex was influenced during the olive oil processing, although the phenol compound and protein bound on the polysaccharide fraction did not increase their molecular weight. However, the use of high temperatures in the citric extraction process from AIS material resulted in a decrease in the molecular weight in all the samples.

### 3.2. Antioxidant Activities of Different Extract

**Figure 2:**
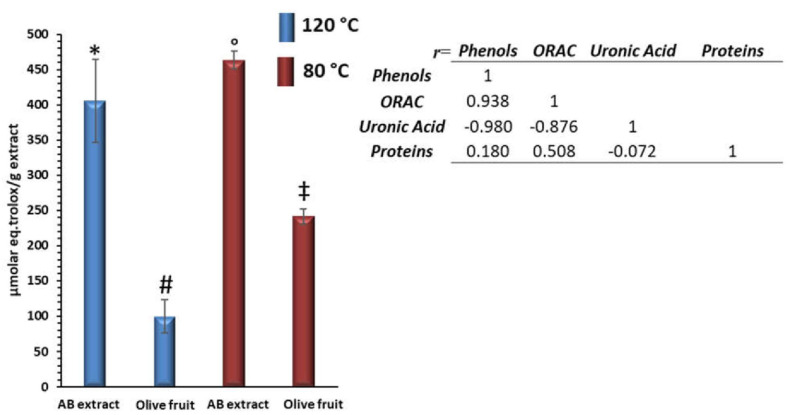
Antioxidant activity of Olive Fruit and AB extracts at 80 and 120 °C. Oxygen radical activity (ORAC) is expressed as μmol Trolox/g extract. Different symbols indicate a statistically significant difference between samples (*p* < 0.05). Each bar is the average value of three replicates. The error bars represent standard deviations (*n* = 3). Pearson’s correlation (r=) of phenols, uronic acid, proteins and antioxidant activities of the extracts was calculated.

The previous study revealed that the pectin-antioxidant extract obtained from alperujo stored in an industrial pomace extractor exhibited a strong in vitro antioxidant effect, with the polyphenol mainly being responsible for their activity; although, once the polyphenols were eliminated, the polysaccharides showed antioxidant activity on their own [16]. In this work, the antioxidant activities of the extracts obtained from fresh olive fruit and the alperujo from the ABENCOR system were studied by the oxygen radical absorbance (ORAC) method (Figure 2). The olive fruit extracts showed the lowest antioxidant activity, between 100 and 241 µmol.eq.trolox/g extract at 120 and 80 °C, respectively. However, the AB extracts presented higher antioxidant activities, with 400 and 460 µmol.eq.trolox/g extract at 120 and 80 °C, respectively. This finding indicated that the antioxidant effect of the extracts was remarkably impacted by the processing of the olives. The higher antioxidant activities determined in AB samples for the two temperatures of extraction might be correlated with the higher contents in polyphenols and protein and lower uronic acids compared to the olive fruit extract. In fact, Pearson’s correlation between ORAC activity and content in phenolic compounds and uronic acids were 0.938 and −0.980, respectively, which indicated that the antioxidant activity was positively correlated with polyphenol content (*p* < 0.05) and negatively correlated with uronic acid content (*p* < 0.05), whereas protein content was not correlated with ORAC (*p* < 0.05). Therefore, this behavior confirmed that the polyphenols associated mainly with acidic pectin substance and a small amount of protein could be one of the main contributors to the antioxidant activity of the extracts, as this interaction was enhanced by the processing.

In the same way that the interactions between the main phenols of the olive (hydroxytyrosol and 3,4-dihydroxy-phenylglycol) and the dietary fiber of strawberry and apple lead to the formation of soluble and insoluble irreversible complexes while maintaining part of their antioxidant activity [31,38], our extracts formed by polyphenols–pectin–protein maintained high antioxidant activity. This indicates a combination of two health-promoting agents, dietary fiber and antioxidants. In addition, like the previous hydroxytyrosol–pectin complex, they were not degraded during the digestion process [39,40], so our extracts should pass through our digestive system unaltered by the enzymatic and pH variations and reach the colon almost intact, where they could be fermented by the gut microbiota and contribute to a healthy colonic environment that is rich in antioxidant compounds.

### 3.3. Effect of the Different Extracts on Cell Proliferation Caco-2 Cells

**Figure 3:**
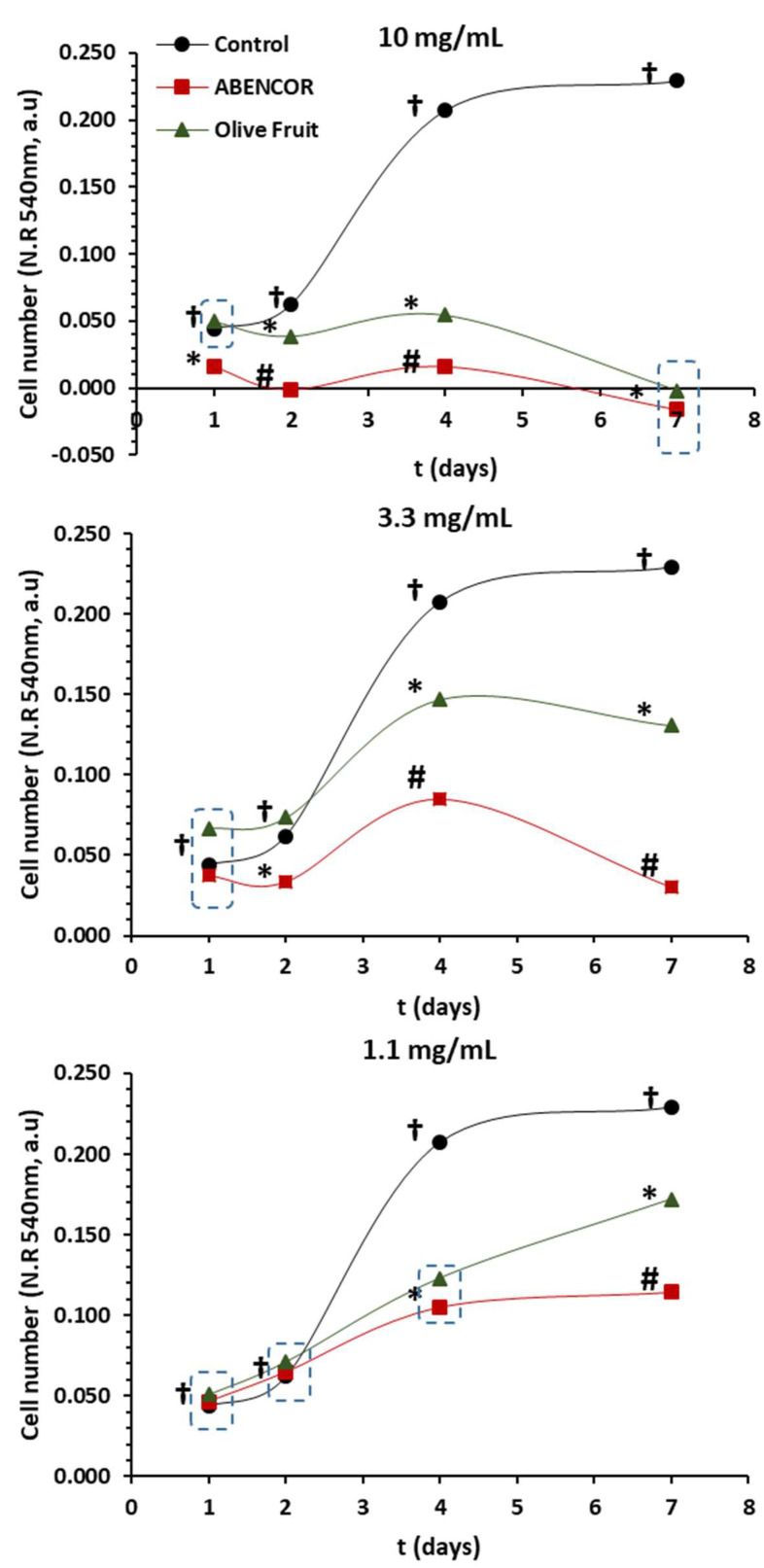
Effect of Olive Fruit and AB extracts at 80 °C on proliferation of Caco-2 cells. Cells (4 × 10^3^ cells/well) were seeded in the presence of increasing concentrations of extracts and allowed to proliferate for one to seven days. Cell number was then estimated by the determination of neutral red uptake. Data represent the average of six replicates; error bars are not shown for clarity. Symbols (*, †, #) indicate a statistically significant difference between extracts and control for each incubation period (one-way ANOVA Tukey test, *p* < 0.05).

The antioxidant activity of the extracts could determine their antiproliferative activity [41]. Since these complex polyphenol–polysaccharide–proteins likely reach the colon almost intact, one of the major targets of their action is gastrointestinal health. In this study, we analyzed the antiproliferative activity of the extracts against the Caco-2 human colon cell line. The results are presented in Figure 3.

Cells were treated for up to seven days and cells were exposed to increasing concentration of the extracts with 1.1, 3.3 and 10 mg/mL of olive fruit and AB extracts obtained at 80 °C. Both extracts showed a concentration-dependent inhibition. However, the olive fruit extract showed a lower effect on Caco-2 cells proliferation, whereas, at 10 mg/mL, the AB extract completely inhibited Caco-2 cells from the first day of treatment; in the olive extract, this was achieved at 7 days. Incubating at 3.3 mg/mL, a high inhibition of cell proliferation was also observed, with significant differences between the extracts. AB was the one with the highest antiproliferative activity. This trend was also observed at 1.1 mg/mL, although the inhibition of proliferation was lower, and the AB extract continued to present the highest antiproliferative activity. The values obtained are once again due to the greater number of polyphenols that this extract presented. These results are in agreement with previous studies by our research group where a direct relationship between a higher antiproliferative activity and their higher antioxidants activity was observed [16]

Although the olive fruit sample had less phenols in its composition, it also had antiproliferative activity at 10 and 3.3 mg/mL. This is in accordance with the data reported by our research group, where extracts obtained from stored alperujo, from which phenols have been eliminated, with a mixture of sodium chlorite and acetic acid presented antiproliferative activity. In any case, the antiproliferative activity of these extracts without phenols was lower than in the cases with phenols [16]. Polyphenols associated with pectic polysaccharides accounted for most of the antiproliferative and antioxidant activities in olive extracts. However, this revealed the importance of the presence of both phenols and polysaccharides in the samples.

## 4. Conclusions

In the present study, we confirmed that an interaction between the polyphenols in the olive fruit and polysaccharides in the cell wall takes place during olive oil processing (milling, malaxation, centrifugation). However, the presence of insoluble-bound phenols into the cell wall matrix of the fresh, unprocessed olive fruit was also found, although in a much lower proportion. Indeed, the interaction of polyphenols with cell wall material was enhanced during olive processing. The extracts obtained from both AIS (fresh olive fruit and ABENCOR system) using conventional pectin extraction were composed mainly of polysaccharides, which constituted the majority of arabinogalactan associated with rhamnogalacturonan, and also phenolic compounds and proteins. The extracts exhibited very promising antioxidant activities and significantly lowered the viability of Caco-2 human colon cancer cells in vitro. The results suggested that the highest contents in the polyphenols in the extracts could be one of the major contributors to their above activities. Therefore, the extracts represent interesting natural compounds that combine the functional and technological properties of dietary fiber and antioxidants, two health-promoting agents, in a unique compound. Further investigation on the digestion, fermentation by the gut microbiota and the intestinal benefits of these pectin–polyphenol complexes will be necessary.

## Figures and Tables

**Table 1:** Yield and chemical composition (% weight) from Olive Fruit and AB extracts at 80 and 120 °C. Values are the mean of 3 replicated ($\bar{x}$) ± SD ($\bar{x}$ = g/100 g lyophilized extract). Different letters in each column indicate statistically significant difference between sample (*p* < 0.05).

		g/100 g Lyophilized Extract					
		Uronic Acid	Phenols	Neutral Sugar	Protein	Ash	Yield
		$\bar{x}$ ± SD	$\bar{x}$ ± SD	$\bar{x}$ ± SD	$\bar{x}$ ± SD	$\bar{x}$ ± SD	%
80 °C	Olive Fruit	38.6 ± 3.5 a	7.0 ± 0.3 a	25.9 ± 1.1 a	1.14 ± 0.03 a	12.5 ± 0.9 a	8.08
	AB extract	21.3 ± 1.8 b	15.5 ± 1.8 b	15. ± 0.7 b	1.9 ± 0.2 b	21.0 ± 1.8 b	5.66
120 °C	Olive Fruit	40.0 ± 7.9 a	5.3 ± 0.5 a	17.3 ± 2.5 bc	2.7 ± 0.2 c	9.7 ± 1.5 c	2.84
	AB extract	31.3 ± 1.4 c	12.0. ± 0.7 c	18.9± 2.1 c	4.3 ± 0.2 d	14.2 ± 0.6 d	5.80

**Table 2:** Sugar composition (% weight and molar ratio) from Olive Fruit and AB extracts at 80 and 120 °C. Values are the mean of 3 replicates ($\bar{x}$) ± SD ($\bar{x}$ = g/100 g lyophilized extract).

	Sugar Composition (g/100 g Lyophilized Extract)							
	80 °C				120 °C			
	Olive Fruit Extract		AB Extract		Olive Fruit Extract		AB Extract	
	$\bar{x}$ ± SD	% molar	$\bar{x}$ ± SD	% molar	$\bar{x}$ ± SD	% molar	$\bar{x}$ ± SD	% molar
Rhamnose	2.5 ± 0.5	3.79	1.8 ± 0.2	4.81	2.1 ± 0.2	3.73	1.5 ± 0.2	3.03
Fucose	0.23 ± 0.05	0.35	0.15 ± 0.01	0.41	0.16 ± 0.01	0.27	0.04 ± 0.06	0.07
Arabinose	13.9 ± 0.2	21.56	5.9 ± 0.2	16.03	2.2 ± 1.9	3.90	1.51 ± 0.08	3.00
Xylose	1.01 ± 0.07	1.57	0.85 ± 0.04	2.31	1.80 ± 0.07	3.15	3.2 ± 0.2	6.45
Mannose	0.77 ± 0.06	1.19	0.67 ± 0.04	1.82	1.00 ± 0.03	1.74	1.5 ± 0.2	2.88
Galactose	5.9 ± 0.1	9.13	2.80 ± 0.08	7.62	7.1 ± 0.1	12.39	4.3 ± 0.9	8.65
Glucose	1.7 ± 0.1	2.60	3.3 ± 0.2	9.05	2.9 ± 0.2	5.05	6.8 ± 0.5	13.56
Uronic acid		59.80		57.94		69.77		62.35

## Data Availability

The data presented in this study are available in the article.
